# Scale-up Chemical Synthesis of Thermally-activated Delayed Fluorescence Emitters Based on the Dibenzothiophene-S,S-Dioxide Core

**DOI:** 10.3791/56501

**Published:** 2017-10-24

**Authors:** Oleh Vybornyi, Neil J. Findlay, Peter J. Skabara

**Affiliations:** ^1^WestCHEM, Department of Pure and Applied Chemistry, University of Strathclyde

**Keywords:** Chemistry, Issue 128, Synthesis, Pd cross-coupling, Buchwald-Hartwig amination, fluorescence, TADF, OLED

## Abstract

We report a procedure to linearly scale-up the synthesis of 2,8-bis(3,6-di-tert-butyl-9H-carbazol-9-yl)dibenzothiophene-S,S-dioxide (compound 4) and 2,8-bis(10H-phenothiazin-10-yl)dibenzothiophene-S,S-dioxide (compound 5) using Buchwald-Hartwig cross-coupling reaction conditions. In addition, we demonstrate a scaled-up synthesis of all non-commercially available starting materials that are required for the amination cross-coupling reaction. In the present article, we provide the detailed synthetic procedures for all of the described compounds, alongside their spectral characterization. This work shows the possibility to produce organic molecules for optoelectronic applications on a large scale, which facilitates their implementation into real world devices.

**Figure:**
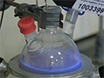


## Introduction

Over the past few decades, researchers have investigated the utilization of organic molecules and their derivatives in organic light-emitting diodes (OLEDs) since their first introduction in 1987.^1^ The discovery of electroluminescence (EL) has led to organic materials finding a wide range of applications in displays and lighting.^2^ Enormous interest in producing new organic compounds and studying their photophysical properties are driven by the advantages of OLEDs over the existing technologies; most notably as they are more energy-efficient and can offer superior device performance whilst maintaining high flexibility, small thickness, and low weight. Nevertheless, in comparison to inorganic light-emitting diodes (LEDs), OLEDs still suffer disadvantages that prevent this technology from large scale commercial production, including shorter lifetimes, and low efficiencies. Until recently, most research was based on first generation OLEDs and emission relied on the fluorescence of the organic materials. Due to the fact that fluorescence can occur only from the singlet state, the maximum theoretical internal quantum efficiency (IQE) of the device is only 25%.^3^,^4^,^5^ By utilizing heavy-metal containing organic complexes (such as Ir, Pt), researchers could overcome the 25% efficiency barrier by harvesting the electrons from both the singlet and triplet states by a radiative transition process.^6^,^7^,^8^ Thus, the maximum theoretical efficiency of second generation phosphorescent OLEDs (PHOLEDs) can be as high as 100%.^9^,^10^ Nowadays, many commercial OLED devices utilize green and red phosphorescent emitting organometallic complexes based on iridium.^11^ Although these materials have gained widespread use in mobile displays and other electronic applications, the cost of the heavy-metal precursor salts and their short lifetimes have pushed researchers to look for alternatives.

There are several objectives that must be achieved for widespread commercialization of OLED technology, such as high stability, efficient power consumption and low production cost of the device. In recent years, it has been shown that thermally activated delayed fluorescence (TADF) emitters can be an effective substitute to PHOLED materials to reach the same IQE value, with these materials now considered as third generation OLED materials.^12^,^13^,^14^ Purely organic TADF emitters can be prepared in a few steps from readily available starting materials making the overall device fabrication commercially attractive to the market. To date, there are several comprehensive literature reviews covering all the aspects from material synthesis to the device fabrication.^15^,^16^,^17^,^18^ The main issue with TADF material preparation is the use of Pd-containing catalysts for the cross-coupling reactions. In order to show the potential for TADF material preparation on an increased scale. we scaled-up the synthesis of TADF emitters from starting materials to the desired products 4 and 5 (Figure 1).^22^,^23^ This detailed protocol is intended to help new practitioners in the field to understand the information associated with the synthesis of TADF materials.

## Protocol

Caution: Please review all relevant material safety data sheets (MSDS) prior to use. Several of the chemicals used in the synthesis are highly toxic and carcinogenic. When working with silica gel, a protective respiratory mask must be worn to prevent particle inhalation. Please observe all appropriate safety rules when performing a chemical reaction including the use of engineering controls (fume cupboard) and personal protective equipment (safety glasses, gloves, lab coat, full length pants, closed-toe shoes).

All reagents and solvents were purchased commercially and were used without any further purification. ^1^H and ^13^C NMR spectra were recorded on a 400 MHz NMR spectrometer using CDCl_3_ as the solvent. Proton NMR chemical shifts are reported as δ values in ppm relative to deuterated solvents: CDCl_3_ (7.26). Data are presented as follows: chemical shift, multiplicity and coupling constant(s) (*J*) are in Hz. Multiplets are reported over the range (in ppm) they appeared. Carbon NMR data were collected relative to the corresponding solvent signals CDCl_3_ (77.16). Melting points were measured using a melting point apparatus. Matrix assisted laser desorption ionisation-time-of-flight (MALDI-TOF) mass spectrometry were run on a Shimadzu Axima-CFR spectrometer (mass range 1-150 000 Da).

### 1. Synthesis of the reported compounds

1. **Synthesis of compound 1 (3,6-di-tert-butyl-9H-carbazole)** 19
  1. Using 500 mL three-neck round-bottom flask, dissolve 9-H-carbazole (10.0 g, 60 mmol) in nitromethane (300 mL) under a nitrogen atmosphere. Then, add zinc (II) chloride (24.3 g, 180 mmol) to this solution.
  2. Pour 2-chloro-2-methylpropane (19.5 mL, 180 mmol) into the 100 mL addition funnel and add dropwise under vigorous stirring.
  3. Stir the resulting mixture at room temperature for 6 h.
  4. Monitor the completion of the reaction by thin layer chromatography (TLC), using dichloromethane: hexane 1:1 v/v as an eluent mixture. After the consumption of 9-H-carbazole, add 100 mL of water to neutralize the solution.
  5. Extract the product three times with 150 mL of dichloromethane each time. Separate the organic layer from the aqueous layer and additionally wash twice with 150 mL of water.
    1. Dry the resulting organic layer with 15 g of magnesium sulfate, and evaporate the solvent under reduced pressure to obtain a grey powder.
  6. Purify the residue by column chromatography using dichloromethane: hexane 1:1 v/v solvent mixture as an eluent provided 13.1 g (79 %) of compound 1 as a white powder. Compound 1 Characterization: ^1^H NMR (400 MHz, CDCl_3_, 25 °C, ppm): 8.12 (d, *J* = 1.9 Hz, 2H), 7.78 (s b, 1H), 7.5 (dd, *J* = 8.5, 1.9 Hz, 2H), 7.33 (dd, *J* = 5.6, 0.5 Hz, 2H), 1.49 (s, 18H). ^13^C NMR (400 MHz, CDCl_3_, 25 °C, ppm): 142.4, 138.2, 123.7, 123.4, 116.3, 110.2, 34.9, 32.2. MS: m/z 279.4 (M+). Melting point: 233-235 °C.
2. **Synthesis of compound 2 (2,8-dibromodibenzothiophene)** 20
  1. Using a 500 mL three-neck round-bottom flask, dissolve dibenzothiophene (15.0 g, 81.3 mmol) in chloroform (100 mL) under nitrogen atmosphere. Next, add dropwise bromine (9.3 mL, 180 mmol) at 0 °C under vigorous stirring.
  2. After complete addition of bromine, raise the temperature to room temperature and stir the reaction mixture for 18 h.
  3. Filter off the resulting white precipitate and repeatedly wash with 50 mL water and then with 15 mL of methanol to isolate 2,8-dibromodibenzothiophene in a yield of 23 g (83%) as a white powder. Compound 2 Characterization: ^1^H NMR (400 MHz, CDCl_3_, 25 °C, ppm): 8.24 (d, *J* = 2.0 Hz, 2H), 7.71 (d, *J* = 8.0 Hz, 2H), 7.58 (d, *J* = 2.0 Hz, 2H). ^13^C NMR (400 MHz, CDCl_3_, 25 °C, ppm): 138.8, 136.3, 130.5, 124.9, 124.3, 118.8. MS: m/z 342.1 (M+). Melting point: 227-229 °C.
3. **Synthesis of compound 3 (2,8-dibromodibenzothiophene-5-dioxide)** 21
  1. Load a 500 mL one-neck round bottom flask with a suspension of 2,8-dibromodibenzothiophene (21.3 g, 60 mmol) and hydrogen peroxide solution (30 mL of 30% (w/w) in H_2_O) in glacial acetic acid (250 mL) and reflux for 2 h under a nitrogen atmosphere.
  2. Cool the resulting mixture to room temperature, and filter off a white precipitate.
  3. Wash three times with 15 mL of water and then 10 mL of methanol, to obtain 19.3 g (85%) of the white powder. Compound 3 Characterization: ^1^H NMR (400 MHz, CDCl_3_, 25 °C, ppm): 8.22 (d, *J* = 1.6 Hz, 2H), 7.70 (d, *J* = 8.0 Hz, 2H), 7.57 (d, *J* = 2 Hz, 2H). ^13^C NMR (400 MHz, CDCl_3_, 25 °C, ppm): 133.3, 130.4, 129.1, 125.6, 124.8, 124.3. MS: m/z 374.1 (M+). Melting point: 360-362 °C.
4. **Synthesis of compound 4 (2,8-bis(3,6-di-tert-butyl-9H-carbazol-9-yl)dibenzothiophene-S,S-dioxide)** 22
  1. Using 500 mL three-neck round-bottom flask, dissolve 2,8-dibromodibenzothiophene-S,S-dioxide (0.45 g, 1.2 mmol) and 3,6-di-tert-butyl-9H-carbazole (0.80 g, 2.86 mmol) in anhydrous toluene (250 mL) and degas by bubbling through nitrogen for 15 min under vigorous stirring.
  2. After this, add tris(dibenzylideneacetone)dipalladium(0) (0.025 g, 0.03 mmol) and 2-dicyclohexylphosphino-2′,4′,6′-triisopropylbiphenyl (XPhos, 0.06 g, 0.12 mmol) to the reaction mixture and degas the solution for another 15 min.
  3. Add sodium tert-butoxide (0.32 g, 0.834 mmol) and tert-butanol (6 mL) and degas the resulting mixture for 15 min.
  4. Heat the reaction solution at 110 °C under nitrogen atmosphere for 18 h.
  5. Cool down to room temperature, add water (150 mL) and extract the organic products into dichloromethane (250 mL) and wash the organic layer twice with 150 mL of water.
  6. Dry the resulting organic layer with 15 g of magnesium sulfate and remove the solvent under reduced pressure to obtain the crude product.
  7. Purify the crude material by column chromatography (using a mixture of solvents as an eluent: dichloromethane: petroleum ether 1:1 v/v) to yield compound 4 as a white solid (0.58 g, 63%). Compound 4 Characterization: ^1^H NMR (400 MHz, CDCl_3_, 25 °C, ppm): 8.13 (d, *J* = 1.6 Hz, 4H), 8.11 (d, *J* = 8.2 Hz, 2H), 7.99 (d, *J* = 1.7 Hz, 2H), 7.82 (dd, *J* = 8.2, 1.8 Hz, 2H), 7.48 (dd, *J* = 8.7, 1.9 Hz, 4H), 7.40 (d, *J* = 8.6 Hz, 4H), 1.45 (s, 36H). ^13^C NMR (400 MHz, CDCl_3_, 25 °C, ppm): 144.4, 144.2, 138.5, 135.8, 133.4, 128.4, 124.4, 124.3, 119.5, 116.8, 109.2, 35.0, 32.2. MS: m/z 769.97 (M+). Melting point: 351-353 °C.
5. **Synthesis of compound 5 (2,8-bis(10H-phenothiazin-10-yl)dibenzothiophene-S,S-dioxide)** 23
  1. Using 250 mL three-neck round-bottom flask, dissolve 2,8-dibromodibenzothiophene-S,S-dioxide (0.67 g, 1.8 mmol) and phenothiazine (0.75 g, 3.77 mmol) in anhydrous toluene (60 mL) and degas by bubbling through nitrogen for 15 min under vigorous stirring.
  2. After this, add tris(dibenzylideneacetone)dipalladium(0) (0.11 g, 0.12 mmol) and 2-dicyclohexylphosphino-2′,4′,6′-triisopropylbiphenyl (XPhos, 0.12 g, 0.23 mmol) to the reaction mixture and degas the solution for another 15 min.
  3. Add sodium tert-butoxide (0.38 g, 3.94 mmol) and degas the resulting mixture for additional 15 min.
  4. Heat the reaction solution to 110 °C under a nitrogen atmosphere for 18 h.
  5. Cool down the reaction mixture to room temperature, add water (150 mL) and extract organic products into dichloromethane (250 mL) and additionally wash twice with 150 mL of water.
  6. Dry the resulting organic layer was with 15 g of magnesium sulfate and remove the solvent under reduced pressure to give the crude product.
  7. Purify the crude product by column chromatography (using solvent mixture as an eluent: dichloromethane: hexane 2:1 v/v) to obtain compound 5 as a white solid (0.9 g, 55%). Compound 5 Characterization: ^1^H NMR (400 MHz, CDCl_3_, 25 °C, ppm): 7.71 (d, *J* = 8.2 Hz, 2H), 7.36 (dd, *J* = 7.7, 1.5 Hz, 4H), 7.28-7.19 (m, 8H), 7.16 (dd, *J* = 7.7, 1.3 Hz, 4H), 7.02 (dd, *J* = 8.1, 1.3 Hz, 4H). ^13^C NMR (400 MHz, CDCl_3_, 25 °C, ppm): 148.8, 141.1, 133.2, 131.8, 129.7, 128.1, 126.9, 125.1, 125.3, 123.1, 121.2, 111.7. MS: m/z 610.08 (M+). Melting point: 353-355 °C.

## Representative Results

The chemical structures of the final TADF emitters (Figure 1) are marked blue for the electron-donating groups and red for the electron-withdrawing core. An alkylated carbazole derivative and a dibrominated dibenzothiopene-S,S-dioxide acceptor molecule can be prepared from inexpensive starting materials (Figure 2). The final products can be prepared by a large-scale version of the Pd-catalyzed amination cross-coupling reaction (Figure 3).

**Figure:**
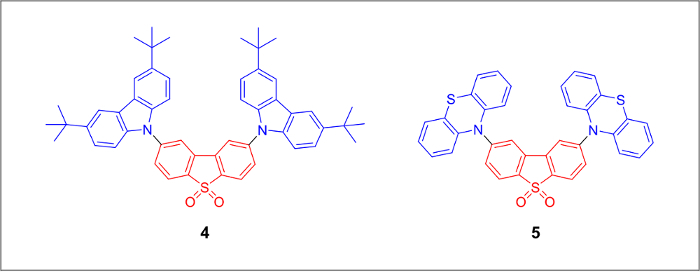

Figure 1**.** Chemical structures of thermally activated delayed fluorescence emitters based on the 2,8-dibenzothiophene-5-dioxide electron acceptor core (shown in red) bearing 3,6-di-tert-butyl-9H-carbazol-9-yl and 10H-phenothiazin-10-yl donor moieties of compound 4 and compound 5 respectively. Electron-donating groups are shown in blue. Please click here to view a larger version of this figure.

**Figure:**
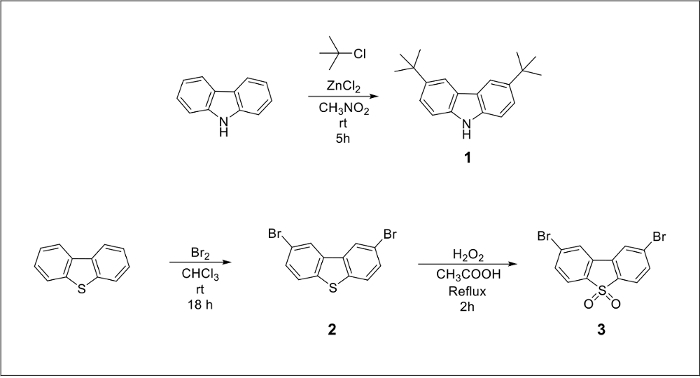

Figure 2**.** Chemical reaction pathways to prepare 3,6-di-tert-butyl-9H-carbazole (compound 1) by alkylation of 9-H-carbazole and 2,8-dibromodibenzothiophene-5-dioxide (compound 3) in two steps starting from commercially available dibenzothiophene. Please click here to view a larger version of this figure.

**Figure:**
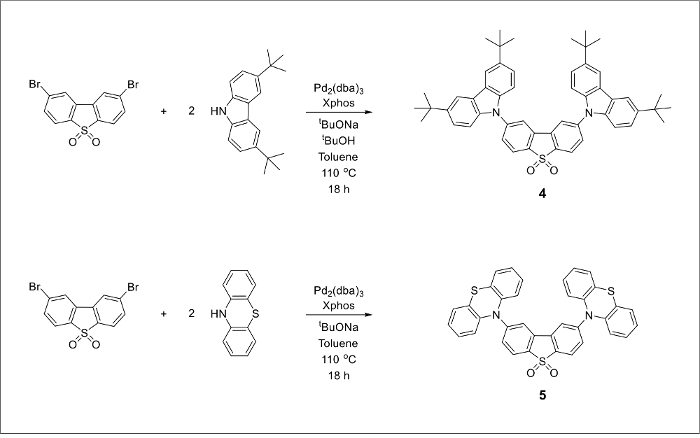

Figure 3**.** Buchwald-Hartwig cross coupling reactions between 2,8-dibromodibenzothiophene-5-dioxide (compound 3) and a corresponding secondary amine to produce compounds 4 and 5. Please click here to view a larger version of this figure.

## Discussion

The purpose of this paper is to show the large-scale synthesis of highly efficient organic materials (Figure 1) for OLED applications from commercially available starting materials. Due to the fact that previous reports only describe the final cross-coupling reaction step and refer to some of the outdated publications, we report the detailed step-by-step synthesis of all the materials together with their full NMR and mass-spectrometry characterization for improved reproducibility of the experiments.

Both emitters bear the same dibenzothiophene-5-dioxide electron acceptor core. The first step in the preparation of compound 3 (Figure 2) is the bromination of the commercially available dibenzothiophene molecule.^20^ The larger scale reaction occurs overnight with a yield of 83%. The resulting dibrominated compound 2 can be further oxidized with a solution of hydrogen peroxide in glacial acetic acid, yielding 19.3 g of a white powder after filtration and washing.^21^ Compound 3 was used in the cross-coupling reaction without any further purification.

The difference in photophysical characteristics between the compound 4 and 5 lies in the electron-donating properties of the amine units. One of the compounds has peripheral alkylated carbazole units while the other has phenothiazine groups. The phenothiazine molecule can be purchased from chemical vendors and used without any modifications. However, in the case of compound 4 tert-butyl groups need to be introduced onto the aromatic ring of the carbazole unit in order to increase solubility in organic solvents. It was reported that in the absence of suitable alkyl groups, the solubility of the product drops, which results in a significantly lower reaction yield.^24^ Alkyl groups can be introduced into each phenyl ring using a Friedel-Crafts reaction between tert-butyl chloride and 9-H-carbazole in the presence of zinc (II) chloride in nitromethane based on the electrophilic aromatic substitution mechanism.^19^ It was found that the purity of the resulting product is not high enough and several by-products are present in the mixture; therefore the crude grey powder must be additionally purified by column chromatography resulting in isolation of 13.1 g of compound 1 recovered as a white powder.

The desired TADF emitters 4 and 5 were synthesized via palladium-catalyzed carbon-nitrogen cross-coupling reactions using previously described literature procedures.^22^,^23^ We found that increasing the amounts of the starting materials does not negatively influence the yield of the desired product, suggesting that further increases to the reaction scale are possible. Compounds 4 and 5 can be obtained in 63% and 55% yield, respectively.

In conclusion, a detailed guideline on how to prepare highly efficient organic molecules for light emitting applications, starting from readily available materials, with the possibility to scale up the whole synthetic procedure has been demonstrated. Through this work, the intention is that large-scale synthesis of many other TADF emitters will ensure a fast transition of the technology from the laboratory bench to industrial applications.

## Disclosures

We have nothing to disclose.
